# Argonaute-dependent small RNAs derived from single-stranded, non-structured precursors

**DOI:** 10.3389/fgene.2014.00172

**Published:** 2014-06-10

**Authors:** Li-Ling Chak, Katsutomo Okamura

**Affiliations:** ^1^Temasek Life Sciences Laboratory, 1 Research Link, National University of SingaporeSingapore, Singapore; ^2^School of Biological Sciences, Nanyang Technological UniversitySingapore, Singapore

**Keywords:** Argonaute proteins, single-stranded RNA, RNA interference (RNAi), gene regulation, RNA modification

## Abstract

A general feature of Argonaute-dependent small RNAs is their base-paired precursor structures, and precursor duplex structures are often required for confident annotation of miRNA genes. However, this rule has been broken by discoveries of functional small RNA species whose precursors lack a predictable double-stranded (ds-) RNA structure, arguing that duplex structures are not prerequisite for small RNA loading to Argonautes. The biological significance of single-stranded (ss-) RNA loading has been recognized particularly in systems where active small RNA amplification mechanisms are involved, because even a small amount of RNA molecules can trigger the production of abundant RNA species leading to profound biological effects. However, even in the absence of small RNA amplification mechanisms, recent studies have demonstrated that potent gene silencing can be achieved using chemically modified synthetic ssRNAs that are resistant to RNases in mice. Therefore, such ssRNA-mediated gene regulation may have broader roles than previously recognized, and the findings have opened the door for further research to optimize the design of ss-siRNAs toward future pharmaceutical and biomedical applications of gene silencing technologies. In this review, we will summarize studies about endogenous ssRNA species that are bound by Argonaute proteins and how ssRNA precursors are recognized by various small RNA pathways.

## INTRODUCTION

Ever since the Nobel prize-winning discovery of RNA interference (RNAi), gene regulation mediated by double-stranded RNAs (dsRNAs) has been one of the most active areas of research in molecular biology (Fire et al., 1998). They demonstrated that only dsRNAs, not antisense or sense single-stranded RNAs (ssRNA) have the ability to induce strong silencing responses in *Caenorhabditis elegans*. Therefore, it appeared that double-stranded structures were prerequisite to the initiation of this mysterious silencing mechanism. Subsequent studies on molecular mechanisms of RNAs clearly showed that silencing is mediated by small RNA species processed from dsRNA trigger molecules (Zamore et al., 2000; Elbashir et al., 2001). Furthermore, the widespread importance of such silencing mechanisms in endogenous gene regulation has also been unveiled (Flynt and Lai, 2008; Castel and Martienssen, 2013; Sun and Lai, 2013). In nearly all eukaryotic organisms, with a notable exception of some budding yeast species (Drinnenberg et al., 2009), endogenous small regulatory RNAs play important roles in a variety of biological processes at transcriptional and post-transcriptional levels.

Small RNA pathways generally involve two key components, RNase III enzymes and Argonaute proteins. RNase III enzymes are dsRNA-specific RNases, and Argonaute proteins are effector proteins that bind to small RNAs and mediate target RNA regulation (Czech and Hannon, 2011). Small RNAs are typically processed from dsRNA precursors by Dicer-class RNase III enzymes as ~20–25 nucleotide (nt) small RNA duplexes, and one of the strands in the small RNA duplex is loaded to the Argonaute protein to form the mature silencing complex. Through the complementarity between small guide RNAs and their target RNAs, Argonaute complexes are recruited to target RNAs to regulate their expression. Therefore, there exist specific pathways recognizing dsRNA molecules to use them as guide RNA precursors.

Because RNase III enzymes are highly selective for particular RNA folding or duplex structures, they can act as gatekeepers of small RNA pathways by distinguishing guide RNA precursors and other cellular transcripts (Court et al., 2013). On the other hand, as discussed below, recent studies have also uncovered a number of Argonaute-dependent small RNA pathways that are initiated by ssRNA precursors with no recognizable structures. How can Argonaute proteins distinguish ss-guide RNA precursors from other cellular transcripts? The goal of this review is to summarize studies regarding mechanisms that selectively load ssRNA-derived small RNAs to Argonaute proteins and discuss common features of ssRNA loading pathways. The fact that mammalian Argonaute proteins retain the ability to load ssRNAs encourages studies on ssRNA-mediated RNAi technologies for future biomedical or pharmaceutical applications. Furthermore, together with our recent realization that RNA modifications and cellular nucleases play specific roles in regulation of RNA metabolism (Sun et al., 2013; Liu and Pan, 2014), findings in ssRNA loading mechanisms may add another dimension to Argonaute-mediated gene regulatory pathways.

## MECHANISMS OF SMALL RNA DUPLEX LOADING

**FIGURE 1:**
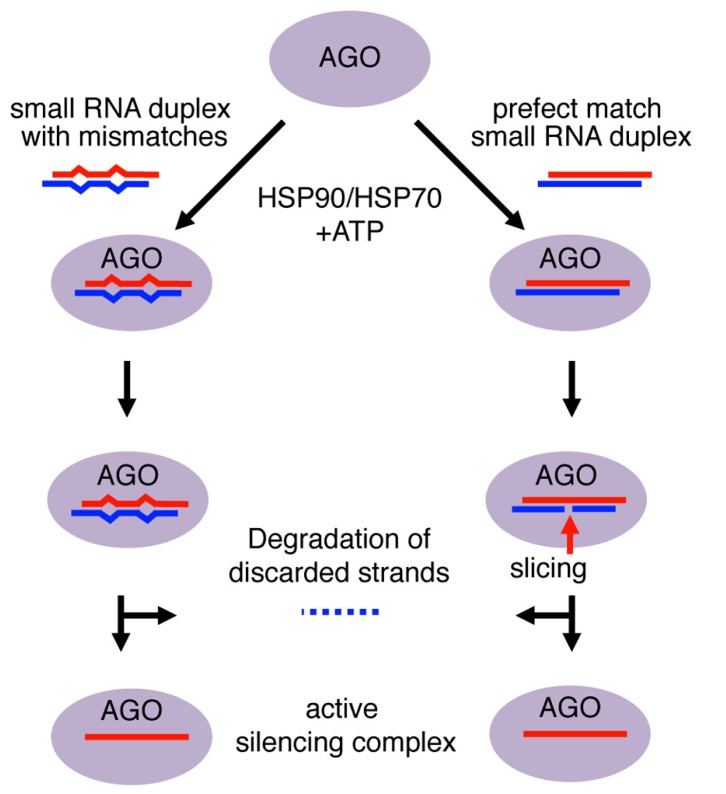
**Mechanism of small RNA duplex loading into Argonaute proteins.** Loading of Argonaute (AGO) with RNA duplexes involves a conformational change in AGO that requires chaperones and ATP. The 5′ end of the guide strand is anchored to the MID domain, while the 3′ end is bound within the PAZ domain. The passenger strand is left unanchored. In small RNA duplexes with mismatches, the energy from the conformational change is used to slowly unwind the duplex and discard the passenger strand. The AGO returns to a close conformation with the guide strand as an active silencing complex. In perfect RNA duplexes, the passenger strand would be sliced in catalytically active AGO and degraded by endonucleases. In contrast, mismatched duplexes would be unwound and the passenger strand would be discarded for subsequent degradation.

Small RNA duplexes are usually produced from longer dsRNA precursors as products of Dicer-class RNase III enzymes (Czech and Hannon, 2011). These Dicer-dependent small RNAs have homogeneous lengths as Dicer enzymes produce small RNA duplexes with specific sizes (e.g., production of ~25–27 nt and ~23 nt products by *Giardia* and *Kluyveromyces* Dicers, respectively; MacRae and Doudna, 2007; Weinberg et al., 2011). The molecular mechanisms of Argonaute loading and duplex unwinding have been mainly studied by biochemical and structural analyses (Kawamata and Tomari, 2010). The small RNA duplex is held in position along the Argonaute RNA-binding groove through selection of the guide strand in the duplex, whose 5′ end is anchored in the MID domain and 3′ end bound by the PAZ domain (Kuhn and Joshua-Tor, 2013). Within the Argonaute complex, the duplex undergoes a maturation step that leaves only the anchored guide strand in the mature silencing complex by discarding the other strand (**Figure 1**).

For small RNA duplex loading, Argonaute proteins are believed to first undergo a conformational change to open the small RNA duplex binding pocket (Kawamata et al., 2009; Yoda et al., 2010). This conformational change allows the Argonaute protein to incorporate the small RNA duplex, which is too bulky for the guide RNA-binding domains of the Argonaute (Kawamata and Tomari, 2010). Consistent with the energy-consuming conformational change, ATP and HSP90/70 are required for duplex loading, although in plants ATP hydrolysis appears to occur after duplex loading (Iki et al., 2010; Iwasaki et al., 2010; Miyoshi et al., 2010). Loaded duplexes undergo either cleavage of passenger strand or duplex unwinding depending on the degree of complementarity, to mature as active silencing complex (Matranga et al., 2005; Miyoshi et al., 2005; Rand et al., 2005; **Figure 1**).

## LOADING OF ssRNAs TO ARGONAUTE PROTEINS *IN VITRO*

The existence of specific loading pathways supported the notion that duplex structures were prerequisite for the formation of active silencing complexes. However, many lines of evidence indicate that Argonaute proteins have the ability to incorporate ssRNA species as guide RNAs. This was first shown by *in vitro* studies using cell lysate systems and by transfection of ss-siRNAs to cultured cells (Martinez et al., 2002; Schwarz et al., 2002; Amarzguioui et al., 2003; Holen et al., 2003). These studies demonstrated that ss-siRNAs could direct target cleavage although ss-siRNA triggers could only induce weak target cleavage activity compared to the duplex siRNA counterparts. Rapid degradation of ssRNA species by cellular RNases presumably may interfere with efficient loading of ssRNAs. Consistent with this idea, short ssRNAs could be efficiently loaded as guide strands when they were mixed with purified Argonaute proteins in the absence of non-specific RNase activity (Miyoshi et al., 2005; Rivas et al., 2005). A study using a human Ago2 protein purified from insect cells revealed an unexpectedly broad range of guide molecules including even a 73 nt non-structured ssRNA (Tan et al., 2009). Consistent with the proposed role for the chaperone machinery in the conformational change of Argonaute to incorporate small RNA duplexes, *in vitro* studies had shown that ssRNA loading could generally occur in a chaperone-independent manner (Miyoshi et al., 2005; Rivas et al., 2005; Tan et al., 2009; Iwasaki et al., 2010).

These *in vitro* studies indicated that a dsRNA structure is not a requirement for guide RNAs to be loaded. Instead, the findings from these studies suggested that the resistance of dsRNAs to ssRNA-specific RNases protects the guide strand from random degradation and allows them enough time to reach Argonaute proteins.

## PROKARYOTIC ARGONAUTE PATHWAYS

Argonaute genes are not specific to eukaryotes, but some prokaryotic genomes encode Argonaute genes with recognizable MID, PIWI and PAZ domains (Makarova et al., 2009). Although the biological roles of prokaryotic Argonautes remain largely unknown, their crystal structures have provided general insights into the molecular activities of Argonaute proteins (Joshua-Tor and Hannon, 2011). A distinctive feature of prokaryotic Argonautes is that at least some of them have higher affinity to ssDNA molecules than ssRNA molecules, and they can use short guide DNAs to cleave RNA targets or even DNA targets (Yuan et al., 2006; Wang et al., 2008, 2009). However, their endogenous guide DNA/RNA molecules were not extensively studied.

**FIGURE 2:**
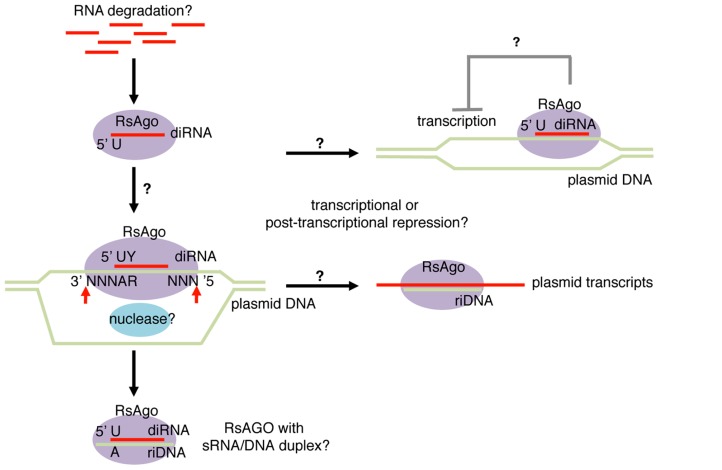
**Bacterial Argonaute-dependent sRNA-sDNA pathway.** Small RNA and DNA fragments bound to RsAgo (*Rhodobacter sphaeroides* Argonaute) were named diRNA (DNA-interacting RNA) and riDNA (RNA-interacting DNA), respectively. These species show enrichment for foreign genes including plasmids, phages, and transposons. diRNAs arise exclusively from sense strand of transcripts and are enriched with uridine (U) at the first nucleotide and pyrimidine (Y) at the second nucleotide. riDNAs are predicted to form duplex structures with diRNAs with 3 nt DNA overhangs at both ends, therefore exhibit adenine (A) and purine (R) enrichment at the fourth and fifth nucleotides counting from the 3′ end. The predicted duplex structures led to the hypothesis that diRNAs guide DNA target cleavage at the positions three nucleotides away from the 5′ and 3′ termini of diRNA. RsAgo represses expression of mRNAs encoded by plasmids by an unknown mechanism transcriptionally and/or post-transcriptionally.

A recent study analyzing Argonaute-associated DNA/RNA molecules in *Rhodobacter sphaeroides* (Rs) identified both DNA and RNA molecules that are associated with the Argonaute protein, RsAgo (Olovnikov et al., 2013). Fifteen to 19 nt RNA and 22–24 nt DNA molecules were recovered from purified Argonaute complexes (**Figure 2**). Similar to many classes of eukaryotic Argonaute-dependent small RNAs, RsAgo-dependent small RNAs showed a strong enrichment for uridine at the 5′ nucleotide. In addition, mild enrichment for pyrimidine was also observed at the second nucleotide counting from the 5′ end. The majority of small RNAs were derived from the sense strand of protein coding or non-coding RNAs, and the levels of small RNAs roughly correlated with the level of their host, long RNA species. However, detailed analysis of individual genes revealed mild depletion of non-coding RNAs and a strong enrichment of RNAs encoded by foreign DNAs such as plasmids, phages and transposons. Interestingly, the authors noted that these foreign DNAs exist as extrachromosomal DNAs at least at certain stages of their lifecycles.

Similarly, short DNAs associated with RsAgo showed enrichment for foreign DNA sequences (Olovnikov et al., 2013). A computational search for predictable short DNA–RNA pairs revealed that DNA–RNA pairs could often be formed with ~3 nt DNA overhangs at both duplex ends. Consistently, adenine and purine were overrepresented at the 4th and 5th nucleotides of the short DNA counting from 3′ ends (**Figure 2**). It is not clear whether both small RNA and small DNA molecules are loaded in RsAgo as guide molecules or they bind to RsAgo as small RNA/DNA heteroduplexes. However, small RNAs were more abundant than small DNAs by ~twofold, which indicated that at least a fraction of RsAgo proteins may form complexes with sRNAs alone.

The enrichment of plasmid-derived sequences suggested that a role for RsAgo is silencing of extrachromosomal DNA such as plasmids. In fact, luciferase reporter assays and quantitative RT-PCR analysis detected elevated gene expression from plasmids in an RsAgo mutant strain while overall expression profiles of host genes were largely unaffected (Olovnikov et al., 2013). Intriguingly, the preferential association of plasmid-derived small RNA/DNA with RsAgo was recapitulated in a heterologous system using *E. coli*. Furthermore, similar preferential loading of plasmid-derived small RNAs was also observed in a previous study with a bacterially expressed yeast Argonaute protein (Nakanishi et al., 2012). These observations suggest that either Argonaute proteins have a conserved intrinsic preference for plasmid-derived sequences, or that *E. coli* retains a mechanism that preferentially load foreign nucleic acids to Argonaute proteins.

So far, there has not been an established link between RNase III enzymes and Ago-dependent pathways in prokaryotic systems. Instead, bacterial RNase III is known to have roles in gene regulation by recognizing structured RNAs, independently of Argonaute activity (Grunberg-Manago, 1999). However, it is noteworthy that RNase III enzymes are involved in another bacterial defense mechanism against foreign nucleic acids, the CRISPR/Cas system (Deltcheva et al., 2011). One interesting possibility is that the link between RNase III and the Argonaute pathway in eukaryotes may have emerged by the combination of the two genome defense mechanisms.

## YEAST priRNAs THAT TRIGGER RdRP-DEPENDENT siRNA PRODUCTION

In *Schizosaccharomyces pombe*, siRNAs play central roles in pericentromeric heterochromatin formation (Castel and Martienssen, 2013). Pericentromeric siRNAs are produced from bidirectional transcription of non-coding RNA loci in a manner dependent on a Dicer (Dcr1), an RdRP (Rdp1), an Argonaute protein (Ago1), and heterochromatin.

**FIGURE 3:**
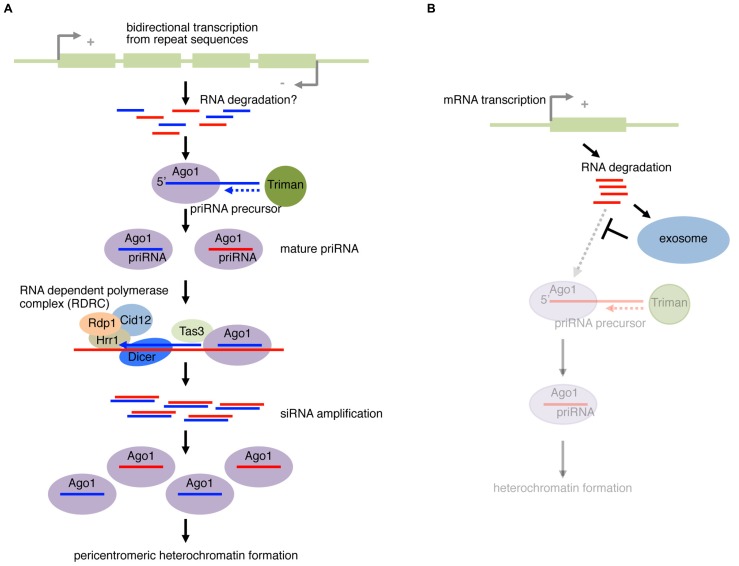
**Fission yeast priRNA pathway. (A)** Bidirectional transcription from pericentromeric repeat sequences produces products that are processed into fragments, possibly through RNA degradation or other unknown mechanism. priRNA (primal RNA) precursor are bound by Ago1 and trimmed on the 3′ end by an exonuclease Triman. The loaded Ago1 with priRNA can find the nascent transcription by RNA Polymerase II in the chromatin, and recruits RNA-dependent polymerase complex (RDRC) containing Rdp1, Hrr1, and Cid12. Dicer cleaves the dsRNA to form siRNA. The siRNA can be loaded to Ago1 that continues the positive feedback cycle. The mature Ago1–siRNA complex recruits factors required for nucleation and spreading of heterochromatin to form pericentromeric heterochromatin. **(B)** priRNAs from mRNAs. In wild-type yeast, the exosome degrades RNA fragments. However, in mutants of exosome components, the priRNA pathway can operate more actively on mRNAs due to the lack of competition with the exosome.

However, the mechanism that initiates the nucleation of RNAi at specific loci is not understood. In a recently proposed model (Halic and Moazed, 2010), a Dcr1-, Rdp1-dependent siRNA amplification cycle is initiated by a small amount of Ago1-loaded RNAs derived from ssRNA fragments of cellular transcripts (**Figure 3A**). The amplification-independent trigger RNAs were named priRNAs (primal RNAs). In agreement with this model, a low level of small RNAs could be detected in mutants of *dcr1* or *rdp1*, in which siRNA amplification is compromised. Small RNA cloning and computational analysis of priRNA showed that priRNAs were generated from a wide range of abundant protein coding/non-coding RNAs in addition to the known siRNA producing loci. However, the majority of reads mapping to protein coding mRNAs were in the sense orientation. priRNAs mapping to mRNAs were strongly enriched in the 3′ downstream regions of annotated protein coding sequences, or the immediate downstream of transcriptional termination sites.

Conversely, a large fraction of antisense priRNAs were mapped to the bidirectionally transcribed *dg* and *dh* loci, where pericentromeric siRNAs are efficiently generated (Halic and Moazed, 2010). Even though generation of priRNAs was detected in both *dg* and *dh* repeats, heterochromatin-independent amplification only occurs with *dg*-siRNAs. Therefore, in addition to bidirectional transcription, there appears to be a mechanism that restricts RdRP-dependent amplification to occur only at the *dg* repeat. These results led to the hypothesis that efficient priRNA production coupled with the amplification mechanism using the bidirectional transcripts from the *dg* locus nucleates the robust RNAi at pericentromeric heterochromatin regions (**Figure 3A**). Consistent with the active roles of priRNAs in heterochromatin formation, *ago1*Δ cells generally showed greater defects in heterochromatin formation at centromeric repeats compared to *dcr1*Δ cells.

If priRNAs are not Dicer products, what determines the lengths of priRNAs? A 3′→5′ exonuclease Triman (Tri1) was identified as an essential priRNA processing factor that trims Ago1-loaded small RNAs (Marasovic et al., 2013). Tri1 is essential for expression of siRNAs and priRNAs at normal levels, and *tri1*Δ cells exhibited defects in priRNA-mediated heterochromatin formation. The remaining siRNAs and priRNAs were slightly longer in *tri1*Δ cells (22–25nt) than in wild-type cells (21–23 nt). In *in vitro* binding assays, Ago1 bound more tightly to 22 nt RNA than longer species, and it was not able to efficiently cleave targets when cleavage was guided by small RNAs ≥ 24 nt in length. Therefore, trimming of small RNAs by Tri1 is essential for their functions. It should also be noted that distinct classes of heterochromatin require distinct set of factors for the siRNA production and heterochromatin formation (Lee et al., 2013b; Yamanaka et al., 2013).

In wild-type yeast, the priRNA pathway and the RNA exosome compete with each other (**Figure 3B**). Therefore, in mutants of exosome components, the priRNA pathway is more active due to the lack of competition (Yamanaka et al., 2013). A complex network of RNAi, heterochromatin and RNA metabolism mechanisms therefore ensures the integrity of gene regulation and chromatin modification (Lee et al., 2013b; Marasovic et al., 2013; Yamanaka et al., 2013).

## ANIMAL piRNA PATHWAYS

A class of metazoan-specific Argonaute proteins, the PIWI family proteins bind a group of 20–30 nt small RNAs collectively called piRNAs (Ishizu et al., 2012; PIWI interacting RNAs). The most well-documented role for piRNAs is defense against transposable elements (TEs) by silencing their expression while they also play roles in gene regulation (Simonelig, 2011). piRNAs are produced from ssRNA precursors by an RNase III-independent mechanism (Vagin et al., 2006; Houwing et al., 2007).

**FIGURE 4:**
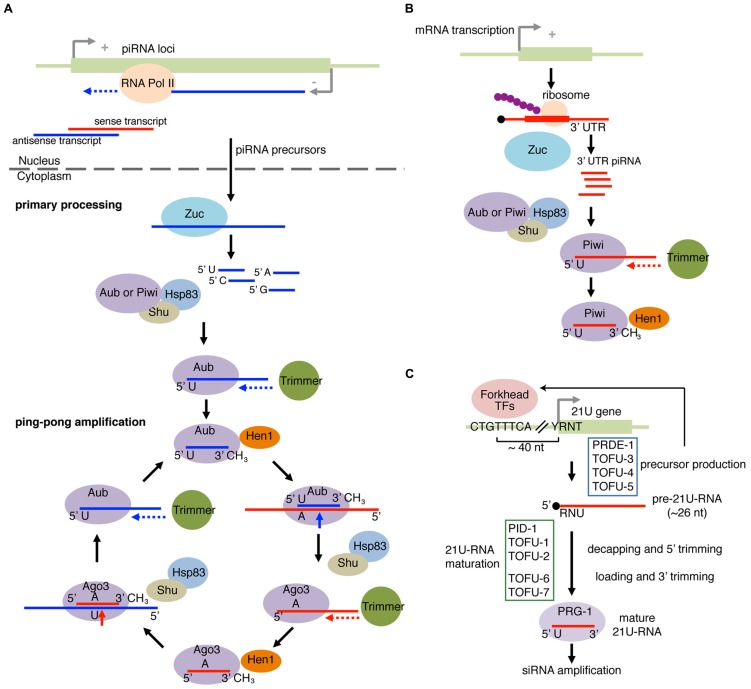
**piRNA pathway and 21U-RNA pathway. (A)** Primary piRNA processing and ping-pong amplification cycle in fly. piRNA precursor transcripts are produced from piRNA loci and are cleaved by an endonuclease Zucchini (Zuc). The PIWI proteins Aubergine (Aub) or PIWI with co-chaperones select the piRNA precursor fragments with enrichment for those with 5′U. The loaded piRNA precursor fragment in PIWI or Aub is then trimmed on the 3′ end by a hypothetical exonuclease Trimmer, and 2′-*O*-methylated at the 2′ position of the 3′ nucleotide by the Hen1 methyltransferase. In the ping-pong amplification pathway, Aub with the mature piRNA targets transcripts bearing the complementary site. The 5′U on the mature piRNA would base pair with an A located on the complementary site. Cleavage occurs 10 nucleotides (inclusive of A) upstream of A, forming the new 5′ end. Shu and HSP83 facilitate the removal of the cleaved 5′ upstream fragment. The cleaved 3′ fragment is then passed to AGO3 and trimmed by Trimmer followed by Hen1 methylation at the 3′ end to form the secondary piRNA/AGO3 complex. This complex recognizes the complementary transcripts from the piRNA clusters and continues the cycle to amplify piRNAs. **(B)** piRNAs from mRNA 3′ UTR. piRNAs from mRNA 3’UTR were found in complexes containing PIWI family proteins. Zuc and Shu are required for production of 3′ UTR piRNAs, suggesting that 3′ UTR piRNAs are recognized by a mechanism similar to the primary piRNA processing pathway. **(C)** 21U-RNA pathway in worm produces piRNAs from the 21U gene. The majority of 21U-RNAs are generated from loci bearing the upstream CTGTTTCA and YRNT motifs (Y = pyrimidine, R = purine, N = any nucleotide). The CTGTTTCA motif is recognized by Forkhead transcription factors to enhance precursor transcription. R corresponds to the transcription start site of the 21U precursor, which is ~26 nt in length. The precursor RNA is loaded to the PIWI ortholog PRG-1. The extra nucleotides at the 5′ and 3′ ends are removed to produce mature 21U-RNAs. 21U RNA can trigger amplification of siRNA to regulate mRNAs and transposons. Recently, factors involved in 21U-RNAs were discovered. Some factors (Forkhead transcription factors, PRDE1, TOFU-3, TOFU-4, and TOFU-5) are involved in precursor biosynthesis, whereas the others (PID-1, TOFU-1, TOFU-2, TOFU-6, and TOFU-7) are involved in processing of 21U-RNA precursors.

The piRNA biogenesis pathway has been most extensively studied in *Drosophila*, and we will describe the mechanism of piRNA biogenesis in the fly system (**Figure 4A**). piRNAs are predominantly derived from the telomeric and pericentromeric regions that house fragmented TE copies (Brennecke et al., 2007). Processing of piRNAs can be roughly divided into two processes known as primary processing and ping-pong amplification (**Figure 4A**). In the primary piRNA pathway, piRNA precursor ssRNAs are loaded to PIWI proteins to generate “primary piRNAs.” An amplification mechanism called the ping-pong amplification uses the primary piRNAs to direct cleavage of complementary TE sense transcripts (Brennecke et al., 2007; Gunawardane et al., 2007). Cleaved TE transcripts are loaded into another PIWI family member, and lead to processing of piRNA precursors into mature piRNAs, therefore forming a feed forward loop (**Figure 4A**). In the fly female gonad, germline cells have both the primary processing and ping-pong amplification pathways, while piRNAs are exclusively produced by the primary processing pathway in gonadal somatic cells (Lau et al., 2009; Li et al., 2009; Malone et al., 2009; Saito et al., 2009).

Processing and loading of piRNAs are believed to take place in specialized perinuclear structures, called Yb-bodies in somatic cells or nuage in germline cells (Lim and Kai, 2007; Olivieri et al., 2010; Saito et al., 2010; Qi et al., 2011). After nuclear transport, piRNA transcripts undergo further processing to mature as 23–29 nt piRNAs. Zucchini (Zuc) was identified as a factor required for piRNA biogenesis (Pane et al., 2007), and was shown to be an ssRNA-specific endonuclease that leaves a monophosphate at the 5′ end of the cleaved product (Ipsaro et al., 2012; Nishimasu et al., 2012). Since piRNAs also possess 5′-monophosphate groups, there is a possibility that Zuc is the enzyme generating 5′ ends of piRNAs. It should be noted that the strong 5′U bias seen in mature piRNAs was not seen with RNA products cleaved by Zuc *in vitro*. Therefore, the 5′ U bias must be introduced by other factors, potentially by PIWI proteins themselves because Argonaute proteins generally have selectivity for particular 5′ nucleotides (**Figure 4A**). In fact, the silkworm PIWI protein that binds primary piRNAs exhibited 5′U preference (Kawaoka et al., 2011; Cora et al., 2014).

HSP90 and its cofactor FKBP were identified as essential piRNA biogenesis factors in mice, silk worms, and flies (Olivieri et al., 2012; Preall et al., 2012; Xiol et al., 2012). While the HSP90 machinery can be involved in the downstream events such as target release (Olivieri et al., 2012; Preall et al., 2012; Xiol et al., 2012), it is possible that the HSP90 machinery is also directly involved in loading of piRNAs. For example, primary piRNAs are reduced in gonadal somatic cells depleted of an FKBP protein Shutdown (Shu) although primary piRNA loading should occur independently of target cleavage by PIWI proteins (Olivieri et al., 2012). Furthermore, the preferred loading of 5′-U species to the primary piRNA PIWI protein was compromised in the presence of an HSP90 inhibitor 17-AAG (17-allylamino-17-demethoxygeldanamycin) in the *in vitro* loading system using silkworm lysate (Izumi et al., 2013). The involvement of chaperone machinery in small RNA loading to PIWI proteins is unexpected because previous *in vitro* studies suggested that ssRNAs could be loaded to Argonaute proteins independently of the chaperone machinery (Miyoshi et al., 2005; Rivas et al., 2005; Tan et al., 2009; Iwasaki et al., 2010). Further molecular analysis will be necessary to elucidate how the chaperone machineries play a role in the piRNA biogenesis pathway.

*In vitro* piRNA loading experiments using silkworm cell lysate also suggested that PIWI proteins are initially loaded with longer intermediate precursors and the loaded RNAs undergo trimming from their 3′ ends by a hypothetical exonuclease named Trimmer (Kawaoka et al., 2011). The presence of 3′ monophosphate or a phosphorothioate linkage could block trimming, indicating that the enzyme is an exonuclease. This activity could only be seen with crude lysate or the pellet fraction after a 1000 × *g* centrifugation, suggesting that the trimmer enzyme is associated with an insoluble structure such as membranes or cytoskeleton. This association has made further biochemical purification and the identification of such enzymes difficult, and the identity of the trimming enzyme has remained unknown to date.

piRNAs are also made from a broad spectrum of protein coding mRNAs (Robine et al., 2009; Saito et al., 2009). A large fraction of genic piRNAs could be mapped to mRNA 3′ UTRs (**Figure 4B**). This enrichment of piRNAs in the 3′ UTR looks similar to the 3′ enrichment of genic priRNAs in *S. pombe* (Halic and Moazed, 2010). This may suggest that 3′ UTRs are fundamentally more susceptible to such ssRNA loading pathways, potentially due to the lack of competition with the translation machinery. Transcriptome-wide analysis showed a poor correlation between the abundance of piRNAs and host mRNAs, suggesting that the piRNA pathway selects a subset of 3′-UTRs, rather than randomly generating piRNAs from all abundant mRNAs (Robine et al., 2009). Genic piRNAs were reduced in *piwi* and *zuc* mutants, but not in mutants of ping-pong amplification components. Therefore, genic piRNAs are produced by the primary piRNA processing pathway. However, it is not known how particular mRNA species are selected for efficient piRNA production.

The 21U-RNA pathway in nematodes is equivalent to the piRNA pathways in other animals. 21U-RNAs are an abundant class of nematode-specific small RNAs and were named after their characteristic 5′ U enrichment and precise 21 nt length (Ruby et al., 2006). The vast majority of 21U-RNAs are derived from two large clusters located on chromosome IV. 21U-RNAs are produced from loci having two upstream motifs: “CTGTTTCA” sequence located at ~40 nt upstream of the first U nucleotide of 21U-RNAs and “YRNT” sequence located just upstream of the 21U-RNA gene with the T in the YRNT motif encoding the 5′U of 21U-RNA (**Figure 4C**). 21U-RNAs are bound by the nematode PIWI ortholog PRG-1, which had been known to be essential for normal fertility (Cox et al., 1998; Batista et al., 2008; Das et al., 2008).

The clusters on chromosome IV are not the only source of 21U-RNAs. Other pol II transcripts starting at the YRNT motif also produce 21U-RNAs at lower efficiencies, even when the locus is not associated with the CTGTTTCA upstream motif (Cecere et al., 2012; Gu et al., 2012). Sequencing analysis suggested that 21U-RNAs are processed from ~26 nt 5′-capped precursors (Gu et al., 2012). The inefficient processing of 21U-RNAs from YRNT loci without the CTGTTTCA motif suggested that additional mechanisms recognizing the upstream motif or other features of genuine 21U-RNA loci might be involved to enhance processing efficiency. Recent studies have identified 21U-RNA biogenesis factors (Cecere et al., 2012; de Albuquerque et al., 2014; Goh et al., 2014; Weick et al., 2014). Based on the levels of accumulation of processing intermediates in mutant animals, 21U-RNA biogenesis factors can be divided into at least two groups. Some factors are involved in production of ~26 nt precursors, and the others are essential for subsequent processing steps (**Figure 4C**).

Analogous to the piRNA pathway in flies and mammals, 21U-RNAs are proposed to play roles in recognition of non-self sequences including TEs, by triggering amplification of a class of siRNAs (22G-RNAs) to establish stable silencing of non-self sequences (Ashe et al., 2012; Bagijn et al., 2012; Luteijn et al., 2012; Shirayama et al., 2012). Furthermore, the strategies of small RNA biogenesis are conserved between the 21U-RNA pathway in nematodes and the piRNA pathway in flies and mammals. Precursor molecules are transcribed from specific genomic loci, and loading of small RNAs is coupled with removal of flanking sequences. Finally, the loaded small RNAs trigger subsequent amplification of small RNAs (**Figure 4**). The strategy also resembles the yeast siRNA pathway (**Figure 3A**), where priRNAs processed from longer ssRNAs trigger siRNA amplification (Halic and Moazed, 2010). The apparent similarity of the strategies may suggest that this is a universal strategy used by many gene silencing pathways, even though the silencing pathways involve seemingly unrelated biogenesis factors (Halic and Moazed, 2010; Czech et al., 2013; Handler et al., 2013; Marasovic et al., 2013; Muerdter et al., 2013; de Albuquerque et al., 2014; Goh et al., 2014; Weick et al., 2014).

## ssRNA-DERIVED SMALL RNAs BOUND BY miRNA-CLASS ARGONAUTES IN ANIMALS

MicroRNAs are a well-studied class of small regulatory RNAs in higher animals (Flynt and Lai, 2008). Many miRNA genes were discovered by computational analysis of small RNA sequencing data combined with RNA folding structure prediction (Berezikov, 2011). Since nearly all known miRNAs are Dicer products, a predictable small RNA duplex structure is usually a requirement for confident miRNA gene annotation (Kozomara and Griffiths-Jones, 2014). Such criteria are essential since small RNA libraries can contain non-functional RNA degradation products and it is difficult to judge whether a small RNA from a non-duplex structure is incorporated in the miRNA pathway. For such small RNAs derived from ssRNAs, further experimental tests such as Argonaute-IP (Immunoprecipitation) analysis would be essential. Nonetheless, accumulating data indicate that miRNA-class small RNAs can be made from precursors without obvious duplex structures.

## DICER-INDEPENDENT BIOGENESIS OF miR-451

To date, *mir-451* is the only verified member of Dicer-independent, Drosha-dependent miRNAs. Although *mir-451* precursor forms a hairpin structure, we will cover this miRNA in this review because the hairpin is not loaded as a duplex and, in theory, miR-451-like small RNAs could be generated in an RNase III-independent manner (see below). *mir-451* was originally found as a conserved hairpin located in a vicinity of a known miRNA *mir-144* and was subsequently shown to associate with Ago2 (Altuvia et al., 2005; Nelson et al., 2007). *mir-451* is widely conserved in vertebrates, and its mutant mice exhibit defects in erythropoiesis (Patrick et al., 2010; Yu et al., 2010).

**FIGURE 5:**
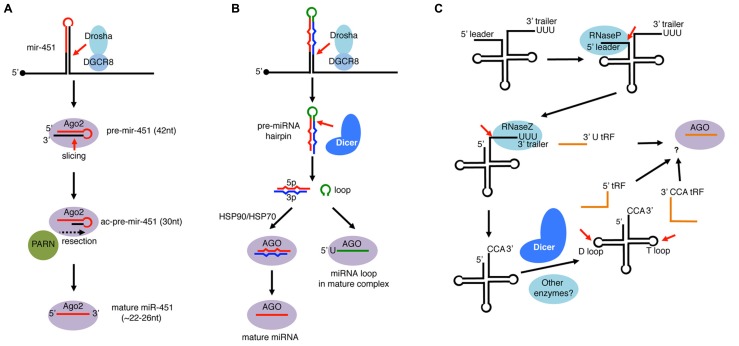
**Single-stranded RNA loading to AGO-clade Argonaute proteins. (A)** *mir-451* is an miRNA widely conserved in vertebrates. Its precursor hairpin is generated by Drosha but too short (42 nt) to be cleaved by Dicer. Instead, the hairpin is directly loaded to Ago2 and cleaved on the 3′ arm by the slicer activity of Ago2. The resulting ac-pre-mir-451 (Ago2-cleaved-pre-miRNA: 30 nt) is further resected by the PARN exonuclease to mature as ~22–26 nt miR-451. **(B)** miRNA loop species loads to AGO. In the miRNA processing pathway, the loop region of pre-miRNA is released as non-structured RNA after processing by Dicer. Processed loops were considered as mere by-products, however, recent studies revealed that select miRNA loops are loaded to Argonaute complexes. Loaded loops are enriched with 5′U species, and their loading occurs independently of the HSP90/HSP70 system, in contrast to mature miRNAs loaded as duplexes. **(C)** Processing of tRNAs in eukaryotes. tRNAs are transcribed by RNA pol III whose transcription terminates with runs of U. The precursor tRNA contains a 5′ leader and a 3′ trailer, which are cleaved by RNaseP and RNaseZ, respectively. Nucleotidyl transferase adds CCA untemplated trinucleotides and the tRNA matures after further chemical modifications and aminoacylation. In addition to mature tRNA products, a variety of small RNA fragments (tRFs) are produced from tRNAs. After RNaseZ-mediated cleavage, some 3′ trailers containing 5′ Us are released as stable species termed 3′ U tRFs. Mature tRNAs can also produce Dicer-dependent species that are cleaved at the D-loop (5′ tRFs) or at the T-loop (3′ CCA tRFs). Some of 3′U tRFs, 5′ tRFs, and 3′ CCA tRFs are loaded to *Argonaute* complexes and play gene regulatory roles.

The *mir-451* hairpin is processed by Drosha–DGCR8 complex similar to canonical miRNAs (**Figure 5A**). However, the resulting hairpin with a 17 bp stem region is too short to be processed by Dicer because processing by Dicer requires > ~22 bp stem regions (Cheloufi et al., 2010; Cifuentes et al., 2010; Yang et al., 2010). Instead of Dicer, the slicer activity of vertebrate Ago2 plays an essential role in *mir-451* processing (**Figure 5A**). Because the *mir-451* hairpin has a highly paired stem region, Ago2 cleaves the 42 nt hairpin at the position complementary to 10th–11th nucleotides from the 5′ end of the hairpin, leaving a 30 nt half-hairpin (ac-pre-miRNA: Ago2-cleaved precursor miRNA).

Although perfect pairing around the cleavage position and an unpaired 5′ nucleotide in the hairpin are important for maturation of *mir-451*-like hairpins, there appears to be no strict sequence restriction (Dueck et al., 2012; Yang et al., 2012). Furthermore, although the natural *mir-451* has a 5′ A, a mutant bearing a 5′ U had slightly enhanced activity against its targets compared to wild-type *mir-451* (Yang et al., 2012). Consistent with the 5′ preference of Ago2 (Frank et al., 2010), their relative activities were observed in the order of U > A > C = G (Yang et al., 2012).

The 30 nt *ac-pre-mir-451* is further resected by an exonuclease PARN to mature as ~22–26 nt species (Yoda et al., 2013). Curiously, a chemically modified *pre-mir-451*-like hairpin that cannot be resected by PARN was nearly as active as the non-modified hairpin in *in vitro* assays. A further attempt was made to verify *in vivo* activity of such resection-resistant hairpins by exploiting an experimental system, where Dicer mutant phenotypes can be rescued by injection of miR-430 (Giraldez et al., 2005). The phenotypes could be partially rescued by a *mir-451*-like hairpin that was reprogrammed to produce mature species having the miR-430 sequence. The degree of rescue by a resection-resistant reprogrammed hairpin was indistinguishable from that of non-modified hairpin through morphological and gene expression analyses (Yoda et al., 2013). Therefore, the removal of tails by PARN from *ac-pre-mir-451*-like molecules is not absolutely essential, although its minor contribution to miR-451 activity cannot be excluded. This is in contrast to the observation with Triman in fission yeast, whose trimming activity was essential for siRNA functions (Halic and Moazed, 2010).

The *mir-451* pathway was recently recognized to be present in flies (Yang et al., 2014). Overexpressed human *mir-451* exhibited regulatory activity against its sensors in flies or fly cell lines. The existence of a variety of non-canonical miRNAs that bypass processing by either Drosha or Dicer raised a question of whether there are miRNAs generated by a completely RNase III-independent mechanism (Yang and Lai, 2011). Although such a gene has not been found in nature, a study has proven that such RNase III-independent miRNAs could be produced in the cell (Maurin et al., 2012). Artificial *mir-451*-like hairpins processed by *Herpesvirus saimiri* snRNAs integrator (Cazalla et al., 2011) or tRNase Z (Phizicky and Hopper, 2010) in a Drosha-independent manner can be processed into mature small RNAs loaded to Ago2 even in Drosha or Dicer knockout cells, providing proof-of-principle. Further studies will be needed to see if the invertebrate *mir-451* pathway and the RNase III-independent Ago2-loading pathway have any endogenous roles.

## LOADING OF miRNA LOOP SPECIES TO ARGONAUTES

In flies and mammals, a selected set of miRNA terminal loops form another source of Argonaute-loaded small RNA molecules (**Figure 5B**). Two recent studies identified miRNA loop species in Argonaute complexes, and demonstrated regulatory activity of overexpressed miRNA loops using luciferase sensor assays (Okamura et al., 2013; Winter et al., 2013). miRNA loops are predicted to be produced as non-structured ssRNA molecules, therefore the loop loading mechanism would be distinct from that of duplex loading. Supporting this notion, *in vitro* recapitulation analysis showed that miRNA loop loading is less sensitive to chaperone inhibitors compared to duplex loading (Okamura et al., 2013).

Consistent with the 5′ U preference of Argonautes, loaded miRNA loop species often have a U at the 5′ end (Okamura et al., 2013; Winter et al., 2013). However, miRNA loop loading efficiency is not solely determined by the 5′ nucleotide. For example, not all 5′U loop species are efficiently loaded, suggesting that additional features of miRNA loops affect loading efficiency. Furthermore, differential Argonaute sorting of loop species was observed in flies (Okamura et al., 2013). Two fly Argonaute proteins, dAGO1 and dAGO2 are known to preferentially bind miRNA and siRNA duplexes, respectively (Czech and Hannon, 2011). siRNA and miRNA duplexes are sorted to the two Argonaute proteins based on the identity of the 5′ nucleotide and the degree of basepairing between the duplex strands (Tomari et al., 2007; Ghildiyal et al., 2010). Therefore, one would expect that ssRNA species bearing the same 5′ nucleotide would be evenly distributed to the two Argonaute complexes. In contrast to this prediction, two 5′U miRNA loop species (*mir-317* and *mir-34* loops) were differentially sorted to dAGO1 and dAGO2. Differential sorting of miRNA loops was recapitulated within *in vitro* loading assays even when the loops were provided as pre-processed synthetic ssRNA oligonucleotides. These results raised a possibility that Argonaute proteins have previously unknown preferences for particular ssRNA species.

These observations challenge a general assumption that binding between guide RNAs and Argonaute proteins is sequence non-specific except for the 5′ nucleotide preference (Kuhn and Joshua-Tor, 2013). However, are Argonaute proteins truly sequence non-specific? A recent large-scale, quantitative study on C5, an RNA binding protein that had been previously believed to have no sequence specificity, revealed that C5 protein actually has a very clear binding preference for particular sequences (Guenther et al., 2013). This points out the fact that the sequence preference of an RNA binding protein cannot be ruled out until the specificity is comprehensively analyzed in a quantitative method.

The ssRNA features that determine Argonaute loading efficiency will be a topic of future research. It also remains to be studied whether and the extent to which loaded loops influence endogenous gene expression. Some of the mammalian Argonaute-loaded loop species have evolutionarily conserved sequences (Michlewski et al., 2008), therefore some of the conserved miRNA loops may have acquired biological roles in gene regulation.

## ARGONAUTE LOADING OF tRNA FRAGMENTS

tRNAs are known to be versatile by playing various roles in gene regulation besides their main function in the translational machinery (Pederson, 2010; Sobala and Hutvagner, 2011). In this section, we will discuss Argonaute-dependent tRNA-fragments (tRFs) that are processed by Dicer-independent mechanisms or generated from precursors with no duplex structures. shRNA-class miRNAs from tRNA precursors have been described; however, these small RNAs will not be covered in this section because these are produced as small RNA duplexes in a Dicer-dependent manner (Babiarz et al., 2008).

tRNA genes are transcribed by RNA polymerase III (RNA Pol III) whose transcription is terminated by a series of Us (**Figure 5C**; Hopper et al., 2010; Phizicky and Hopper, 2010). pre-tRNAs are then processed by RNase P to remove the 5′ leader sequence (Walker and Engelke, 2006). The 3′U trailer sequence is removed by RNase Z and non-templated CCA nucleotides are added to the trimmed 3′ end by tRNA nucleotidyl transferase. The processed tRNA undergoes further modification steps to mature as functional aminoacylated tRNAs.

There are three major classes of Argonaute-dependent tRFs: 3′ CCA tRFs, 3′U tRFs, and 5′ tRFs. 3′ CCA tRFs and 3′U tRFs are produced from the 3′ ends of mature and pre-tRNAs, respectively (Kawaji et al., 2008; Haussecker et al., 2010; Li et al., 2012; Maute et al., 2013; **Figure 5C**). While at least some 3′ CCA tRFs are processed by a Dicer-dependent manner (Cole et al., 2009), 3′U tRFs are generated by RNase Z-mediated cleavage. Interestingly, a 3′U tRF species, Cand45 appeared to be preferentially loaded to Ago3 and Ago4 when Argonaute proteins were individually overexpressed (Haussecker et al., 2010), raising a possibility that mammalian Argonautes have preferences for particular ssRNAs.

A 5′ tRF, tRNA-Gln is abundant in HeLa cells and is produced in a Dicer-dependent manner (Cole et al., 2009). However, size exclusion chromatography analysis and Argonaute-IP assays suggested that the majority of 5′ tRF tRNA-Gln was not loaded (Cole et al., 2009), therefore not all tRFs are loaded to Argonaute. tRNA-Gln small RNA fragments were beta-elimination resistant, indicating that there was a chemical modification of the tRNA-Gln 5′tRF at the 3′ nucleotide. It is possible that the 3′ modification is a cause of the inefficient association between the tRNA-Gln 5′tRF and Argonaute proteins, because previous structural studies have revealed that the PAZ domain of human Ago1 has a higher affinity to short RNAs with 2′-, 3′-OH groups at the 3′ nucleotide (Ma et al., 2004).

5′ tRFs and 3′ CCA tRFs were also found in Argonaute complexes in other organisms, such as Argonautes in *Arabidopsis* (Loss-Morais et al., 2013). Twi12, which is a PIWI protein essential for growth in *Tetrahymena* has been reported to bind 3′CCA tRFs (Couvillion et al., 2009, 2010). Although various mechanisms are involved in tRNA-mediated gene regulation, ssRNA loading appears to be a part of tRNA-mediated gene regulation.

## ARGONAUTE-BOUND SMALL RNAs FROM TRANSCRIPTION TERMINATION SITES

A comprehensive study of small RNAs recovered in immunopurified Argonaute complexes from human cells also revealed a class of small RNAs mapping to the sense strands of 3′UTRs, named transcription termination site-associated (TTSa) RNAs (Valen et al., 2011). While TTSa RNAs lack evidence for precursor duplex structures, they were strongly enriched in the Argonaute-IP libraries and showed a characteristic size peak at 22–23 nt. There is an enrichment at the 3′ ends of 3′ UTRs suggesting the connection between Argonaute loading machineries and mRNA 3′ processing mechanisms. Their biological roles and precise mechanisms of biogenesis remain to be elucidated (Valen et al., 2011).

In summary, a variety of endogenous ssRNAs are selectively loaded to miRNA-class Argonautes in animals. The mechanisms of precursor selection and the biological roles for such ssRNA-derived small RNAs will be an interesting topic of research.

## COMMON FEATURES OF ssRNA LOADING PATHWAYS

To maintain the integrity of gene regulatory networks, Argonaute proteins should not randomly sample all cellular transcripts to generate regulatory RNAs. The mode in which guide RNAs are selected in a majority of eukaryotic Argonaute pathways is through usage of dsRNA molecules as guide RNA precursors (Okamura, 2012). However, as discussed above, although a variety of mechanisms are involved in the recognition and loading of ssRNA precursors, there are common features of ssRNA loading mechanisms.

3′ trimming of loaded sRNA precursors is commonly seen in ssRNA loading pathways. 3′ trimming is essential for some Argonaute proteins (e.g., *S. pombe* Ago1; Marasovic et al., 2013) whereas longer guide RNAs can efficiently mediate functions of others (e.g., mammalian Argonautes; Yoda et al., 2013). Although the size of resulting mature small RNA species appears to be determined by the size of the RNA region protected by the Argonaute protein, it is interesting that specific exonucleases, not general RNases, have roles in trimming of small RNAs. In fact, knockdown of Nibbler, the 3′→5′ exonuclease responsible for 3′-trimming of dAGO1-loaded miRNAs derived from canonical miRNA duplexes (Han et al., 2011; Liu et al., 2011), surprisingly enhanced 3′-trimming of overexpressed miR-451 in fly cells. This suggested that Nibbler competes with the enzyme trimming ac-pre-mir-451 species (Yang et al., 2014). There are specific exonucleases playing major roles in individual pathways, and future studies should uncover the complex network of exonuclease-mediated regulation of the small RNA pathway.

In addition, mechanisms that prevent random ssRNA fragments from fortuitous ssRNA loading are often seen. In the *S. pombe* RNAi pathway, aberrant RNA molecules are targeted by both RNAi and exosome pathways (Lee et al., 2013b; Marasovic et al., 2013; Yamanaka et al., 2013; **Figure 3B**). In *Arabidopsis*, mutations in 5′→3′ exoribonucleases or a nucleotidase/phosphatase result in the accumulation of miRNA loops. It has not been tested whether the accumulated loops are loaded to Argonautes, but the results suggested that there are active mechanisms removing miRNA loops after processing at least in plants (Gy et al., 2007). Presumably, the rates of general RNA turnover are controlled in a tissue-specific manner and would vary depending on cellular conditions. In addition, the pharmacological use of modified nucleotide analogs can potentially affect stability of cellular RNAs. 5-Fluorouracil, a widely used chemotherapeutic agent for solid tumor is known to inhibit RNA degradation by the exosome (Kammler et al., 2008). Therefore, further research is encouraged to better understand whether and how RNA metabolism pathways affect gene expression via small RNA pathways in normal and disease settings.

## RNAi TECHNOLOGIES MEDIATED BY ssRNAs

Despite their promising capability, ds-siRNA mediated RNAi technologies have limitations. Delivery of ds-siRNA duplexes to the target tissue has been a greater challenge compared to the more feasible delivery of ss-oligonucleotides (Bennett and Swayze, 2010). Furthermore, as it is difficult to completely eliminate loading of the sense strand of ds-siRNAs, siRNA duplexes would have a higher risk of off-target effects by the contribution of sense strands in ds-siRNAs. Given that mammalian Argonautes have the ability to incorporate ssRNAs as guide RNAs, a better solution could be to use ss-siRNA triggers that are stable enough to be efficiently loaded to Argonaute proteins *in vivo*.

**FIGURE 6:**
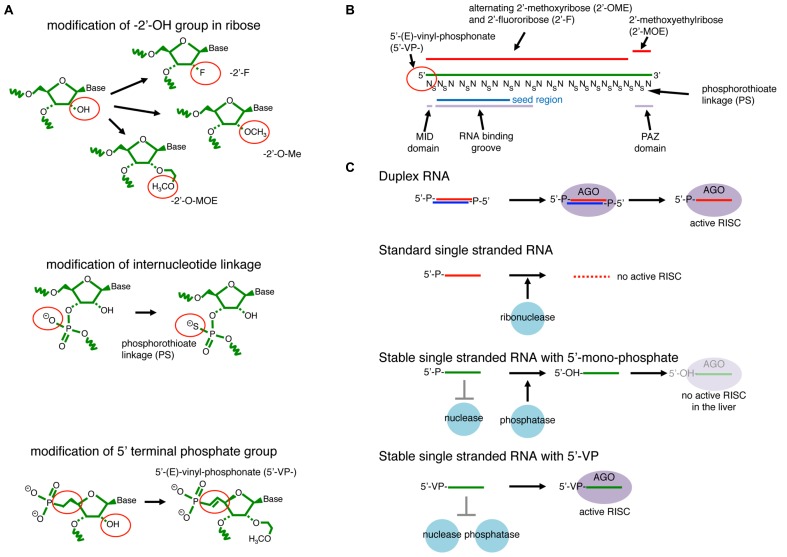
**RNAi with modified single-stranded- (ss-) siRNAs. (A)** Examples of RNA modifications that stabilize ssRNAs. The 2′-OH group on ribose is modified to have 2′-fluoro (-2′-F), 2′-*O*-methyl (-2’O-Me), or 2′-methoxyethyl (-2′-MOE). The ss-RNA backbone contains either phosphodiester or phosphorothioate (PS) internucleotide linkages. **(B)** An example of effective ss-siRNA. The ss-siRNA used in Lima et al., (2012) contains 5′VP, 2′F, 2′-*O*-Me, and 2′-MOE modifications at specific positions. PS linkages are also introduced to all linkages between the first two nucleotides and last eight nucleotides, and also between every two nucleotides from the 3rd to 14th positions. The more stable 5′-(*E*)-vinylphosphonate (5’VP) share similar chemical properties to 5′-phosphate, which is essential for recognition by the Argonaute MID domain. The backbone of nucleotide position 2 to 10 runs along the RNA-binding groove. The last two nucleotides, positions 20 and 21, interact with the Argonaute PAZ domain. **(C)** Duplex RNAs are resistant to abundant cellular nucleases that are usually ssRNA specific. They are able to form the mature silencing complex through the canonical duplex loading pathway. ssRNAs are highly unstable in cells and not able to efficiently form the silencing complex. Nuclease-resistant ssRNAs modified at their 2′ positions and/or linkages are stable in cells but susceptible to cellular phosphatases that remove the 5′ phosphate. Therefore, they do not effectively form the silencing complex. A combination of 5′VP and 2′-linkage modifications blocks degradation and dephosphorylation, and ssRNAs with such modifications are stable enough in the cellular environment to directly form mature silencing complex.

To achieve this, a major strategy is to avoid RNase-mediated degradation by replacing the 2′-OH group or the phosphodiester-backbone linkage with other chemical modifications (**Figure 6A**). However, care has to be taken as Argonaute proteins have many direct contacts with RNA molecules including hydrogen bonds with 2′-OH groups (Kuhn and Joshua-Tor, 2013). ssRNAs with boranophosphate linkages at particular positions show enhanced RNAi activity compared to the unmodified ssRNA counterpart with elevated resistance against RNases (Hall et al., 2004). However, when the effects of this modification at different positions were compared, the stability of modified ssRNAs and their efficacies did not show a clear correlation. This suggested that RNAi efficacy is not determined only by RNA stability but there are other important factors such as the binding affinity of modified RNAs to Argonaute proteins or the capability of guiding cleavage. Another study comparing a panel of 2′-modifications showed that 2′-fluoro-modification could increased RNAi activity in cultured cells as well as mouse animals compared to 2′OH-ssRNA triggers (Haringsma et al., 2012).

A recent study demonstrated that activity of ssRNA-mediated RNAi in mouse animals could be greatly enhanced by introducing a combination of modifications to ssRNA molecules (Lima et al., 2012). The study found that RNA oligos with mixed 2′-Fluoro- (2′-F-) and 2′-O-metyl- (2′O-Me-) modified nucleotides with uniform phosphorothioate- (PS-) linkages in the 3′ half enhanced the ss-siRNA activity in cultured cells (**Figure 6B**). The introduction of a PS-linkage after the first nucleotide and 7 PS-linkages in the next 12 nt followed by uniform PS-modifications in the 3′ region further enhanced the stability of ssRNAs without impairing target cleavage activity (**Figure 6B**). On the other hand, ssRNAs with uniform PS modifications in the 5′ half could not induce silencing, consistent with the tight and specific interaction between the 2nd and 10th nucleotides of the guide strand and the RNA binding groove of Argonaute (Kuhn and Joshua-Tor, 2013).

However, this combination was still not effective enough for strong knockdown in mouse animals (Lima et al., 2012). Mass-spectrometry analysis revealed that 5′ monophosphates could be removed from the introduced ss-siRNAs in the mouse liver as quickly as 6 h after injection. A screen for more stable 5′-modifications identified 5′-(*E*)-vinylphosphonate (5′VP), which is more stable but has chemically similar properties to the natural phosphate (**Figure 6C**). The study demonstrated that efficient knockdown could be achieved *in vivo* and efficient gene silencing could be observed for at least 3 days after a single injection of ssRNAs chemically modified by this formulation (Lima et al., 2012).

Encouraged by the success of efficient gene knockdown by ss-siRNAs in the whole animal, ss-RNAi has been tested in an animal disease model for Huntington’s disease (HD; Yu et al., 2012). HD is characterized by progressive neurodegeneration that is caused by an expansion of the CAG repeat in the Huntingtin (HTT) coding region in HD patients (Lee et al., 2013a). An ideal clinical approach is to inhibit the expression of mutated HTT allele while not affecting the wild-type allele. A panel of ss-siRNAs against the CAG repeat with mismatches at different positions was tested for knockdown efficiency and selectivity to the mutant allele. One such ss-siRNA achieved > 30-fold selectivity for the mutant allele with a high potency in a cell line derived from an HD patient. The same ss-siRNA could selectively reduce the expression of mutated HTT protein in the brain of HD model mouse when the ss-siRNA was continuously infused into the cerebral spinal fluid.

More recently, ss-siRNAs against another gene containing CAG repeats, ATX-3 (ataxin-3), were tested for the ability to selectively silence the expanded allele (Liu et al., 2013). The expansion of CAG repeats in the ATX-3 gene causes Machado-Joseph disease. This study again could identify highly selective and effective ss-siRNAs using a panel of chemically modified ss-siRNAs containing mismatches in different positions. In addition to the reduction of ATX-3 protein from the expanded allele, a shorter protein isoform was observed by Western blotting when some of the ss-siRNAs were transfected. The shorter species corresponded to an alternative splicing isoform that skipped the exon containing the CAG repeat. This resembled the exon skipping observed with antisense peptide nucleic acids (PNAs) that cannot be incorporated in Argonaute complexes (Liu et al., 2013). Therefore, the results suggested that chemically modified ss-siRNAs can function through both Argonaute-dependent and -independent mechanisms, and precise control of their mode of action can be a future challenge.

These studies demonstrated the potential of chemically modified ss-siRNAs for animal knockdown experiments and opened a possibility to use such modified RNA molecules for drug development. By combining the power of advanced nucleic acid chemistry and structural information of Argonaute complexes, it would probably be possible to further improve ss-siRNA design in more directed ways. Furthermore, it will be interesting to see whether there are any natural chemical RNA modifications that promote or inhibit Argonaute loading. Although some of the modifications used for ssRNA-mediated RNAi have not been seen in natural RNA molecules, it has been recently recognized that cellular RNAs are often subjected to various chemical modifications (Machnicka et al., 2013). Future studies may discover links between RNA modifications and ssRNA loading pathways.

## CONCLUSIONS AND FUTURE PERSPECTIVES

As described above, ssRNA loading pathways were generally revealed as atypical activity of Argonaute proteins in previous studies in eukaryotes. However, the importance of ssRNA loading pathway in unicellular organisms and the common features of ssRNA loading pathways suggest that the ability of eukaryotic Argonautes to incorporate ssRNAs as guide molecule is a universal activity that was inherited from the primordial ancestral Argonaute protein. Because previous studies were focused on roles for RNase III products in Argonaute-mediated gene regulatory pathways, biological significance of ssRNA products may have been overlooked. Therefore, it remains to be seen in future studies how many important ssRNA-derived small RNAs exist in higher organisms.

Furthermore, the finding that particular ssRNA species can be selectively loaded to individual Argonautes raised new questions. How do Argonautes select small RNAs? Do they have sequence specificity, and if so, does the sequence specificity of Argonautes affects stability of guide RNA-Argonaute complexes or efficiency of loading even when the guide RNAs are derived from duplexes? Further molecular and structural biology studies will be needed to answer these questions.

Sequence preference of distinct human Argonaute proteins (Haussecker et al., 2010) is particularly interesting because it would be ideal to direct artificial siRNAs to the slicer Argonaute, Ago2, to maximize efficacy and minimize off-target effects. Future studies of ssRNA loading machineries may open additional possibilities for improvement of ss-RNAi technologies.

## Conflict of Interest Statement

The authors declare that the research was conducted in the absence of any commercial or financial relationships that could be construed as a potential conflict of interest.
